# Fabrication of an electrochemical sensor based on magnetic molecularly imprinted polymer for detection of sunset yellow dye

**DOI:** 10.1038/s41598-026-38556-x

**Published:** 2026-02-11

**Authors:** Sumeet Malik, Waqas Ahmad, Adnan Khan, Gul Rahman, Sabir Khan, Maria Del Pilar Taboada Sotomayor, Thiago Machado da Silva Acioly, Mohd Abul Hasan, Alibek Ydyrys, Muhammad Ilyas

**Affiliations:** 1 Institute of Chemical Sciences, University of Peshawar, 25120 Khyber Pakhtunkhwa, Pakistan; 2 Center for Chemical, Pharmaceutical and Food Sciences, Federal University of Pelotas, Pelotas, RS Brazil; 3 Graduate Program in Materials Science and Engineering (PPGCEM), Technological Development Center, Federal University of Pelotas (UFPel), Pelotas, Brazil; 4 Institute of Chemistry, São Paulo State University (UNESP), Araraquara, Brazil; 5 Federal Institute of Education, Science and Technology of Sertão Pernambucano (IFSertãoPE), Campus Floresta, Floresta, 56400-000 Pernambuco Brazil; 6 Department of Civil Engineering, College of Engineering, King Khalid University, Abha, Kingdom of Saudi Arabia; 7 Biomedical Research Centre, Al-Farabi Kazakh National University, Almaty, Kazakhstan; 8 Khoja Akhmet Yassawi International Kazakh-Turkish University, Sattarkhanov Ave. 29, Turkistan, 161200 Kazakhstan; 9 Department of Environmental Sciences, Faculty of Sciences, International Islamic University, Islamabad, Pakistan; 10 Postgraduate Program in Animal Science (PPGCA), State University of Maranhão (UEMA), São Luís, Maranhão, 65080-400 Brazil

**Keywords:** Magnetic molecularly imprinted polymer, Sorption, 1-vinylpyridine, Azo dye, Voltammetry, Chemistry, Environmental sciences, Materials science, Nanoscience and technology

## Abstract

Sunset Yellow (SY) is a widely used synthetic azo dye in the food, pharmaceutical, and cosmetic industries, but its excessive release poses serious health and environmental risks. In this study, a magnetic molecularly imprinted polymer (MMIP)-based electrochemical sensor was developed for the selective detection of SY using 1-vinylpyridine as the functional monomer. Scanning electron microscopy (SEM) revealed irregular particle morphologies with an average diameter of approximately 69 nm, attributed to surface cavities formed during the imprinting process. Batch sorption experiments confirmed the high specificity of the MMIPs, with a maximum sorption capacity of 80 mg g^− 1^ under optimal conditions (pH 2, sorbent dosage 2 mg, contact time 18 min). Sorption kinetics followed a pseudo-second-order model, and adsorption behavior was best described by the Langmuir isotherm, indicating monolayer adsorption. Electrochemical measurements demonstrated that the fabricated sensor exhibited high sensitivity, selectivity, and stability for SY detection. The sensor performed optimally at pH 7, an accumulation time of 60 s, and a concentration of 1.5 × 10⁻³ M, with recovery values of 72.9–99.3% in real water and beverage samples, highlighting its practical applicability for environmental and food analysis. The LOD and LOQ values were found to be 5.82 × 10^− 5^ M and 1.76 × 10^− 4^ M, respectively. These results confirm the effectiveness of MMIP-based platforms for rapid, accurate, and selective monitoring of synthetic dyes in complex matrices.

## Introduction

Rapid industrialization driven by population growth and increasing lifestyle demands has significantly affected the quality of air, water, and soil. To enhance product appeal and market competitiveness, industries such as textiles, leather, cosmetics, pharmaceuticals, and food processing heavily depend on dyes and coloring agents in their production processes^1,2^. SY FCF (E110), an azo dye, is one of the most widely used colorants in foods and beverages due to its excellent water solubility and stability over a wide pH range^3^. However, despite its broad use, SY has been associated with several adverse health and environmental impacts. Although conclusive evidence is limited, studies have linked it to genotoxic effects, allergic reactions, and potential connections to hyperactivity in children. Its safety continues to be evaluated by regulatory bodies such as the FDA and EFSA. Due to its poor biodegradability, this dye tends to persist in aquatic ecosystems, posing long-term ecological risks^4,5^.

Several methods have been explored for dye removal, including oxidation^6^, photocatalysis^7^, biodegradation^8^, membrane filtration^9^, and sorption^10^. Among these, sorption using advanced adsorbents remains one of the most effective and straightforward approaches. However, conventional adsorbents often suffer from low selectivity and high cost. Consequently, molecularly imprinted polymers (MIPs) have emerged as promising synthetic materials owing to their high selectivity, chemical stability, and cost-effective preparation. Their performance can be further enhanced by reducing MIPs to the nanoscale or integrating them with magnetic nanoparticles to form magnetic molecularly imprinted polymers (MMIPs)^11^. These hybrid materials exhibit improved binding performance, greater surface area, and efficient separation. MMIPs combine the molecular recognition ability of MIPs with the magnetic responsiveness of nanoparticles such as Fe_3_O_4_ or γ-Fe_2_O_3_. While the polymeric shell provides specific binding sites, the magnetic core enables rapid and simple recovery^12^.

MMIPs have gained increasing attention in electrochemical sensing applications due to their facile magnetic separation, excellent specificity, and reusability. Typically, an electrochemical sensor consists of three main components: a transducer (commonly an electrode that converts chemical interactions into electrical signals), a recognition element (in this case, the MMIPs), and a signal processor, which interprets the output response^13,14^. MMIP-based sensors are particularly effective for detecting food additives such as SY in complex real-world samples, offering fast response times, high sensitivity, and low detection limits.

The novelty of this work lies in the design of a magnetic molecularly imprinted polymer-based electrochemical sensor for the selective detection of SY, providing a cost-effective, sensitive, and eco-friendly approach to monitoring synthetic dyes in environmental samples. This approach contributes to the achievement of several United Nations Sustainable Development Goals (SDGs), including SDG 3 (Good Health and Well-being), SDG 6 (Clean Water and Sanitation), SDG 9 (Industry, Innovation and Infrastructure), SDG 11 (Sustainable Cities and Communities), SDG 12 (Responsible Consumption and Production), and SDG 14 (Life Below Water), by promoting sustainable, low-cost, and efficient strategies for the detection and removal of hazardous dyes from water systems. To the best of our knowledge, this is the first report of an MMIP-based electrochemical sensor specifically designed for SY quantification in environmental samples, highlighting its novel contribution to both analytical chemistry and environmental sustainability.

## Materials and methods

### Materials

SY dye (90%, MACSEN LABS), 1-vinylpyridine (95%, USA Chemical Suppliers), ethylene glycol dimethacrylate (EGDMA, 99%, Sigma Aldrich), azobisisobutyronitrile (AIBN, 98%, Sigma Aldrich). All reagents were of analytical grade.

### Preparation of Core-Shell MIPs

The initial stage of the polymerization process involved preparing a suitable core material to support subsequent polymer growth. Magnetic nanoparticles (MNPs) were synthesized using a co-precipitation technique^14^. In 80 mL of deionized water, a 1:2 molar ratio of iron (II) sulfate and iron (III) chloride (1.72 g and 2.32 g, respectively) was dissolved. Nitrogen gas was continuously purged through the solution to maintain an inert atmosphere. After homogenization, the reaction mixture was heated to 80 °C and stirred for 30 min before ammonium hydroxide was gradually added to initiate the co-precipitation process^15^.

The successful synthesis of MNPs was indicated by the formation of black precipitates, which were collected using an external magnet, thoroughly washed to remove impurities, and vacuum-dried. The MNP surface was modified in two steps to introduce vinyl functional groups to facilitate polymerization. First, 300 mg of MNPs were sonicated for 15 min in a solution containing 40 mL of ethanol and 4 mL of deionized water. Then, 5 mL of ammonium hydroxide and 2 mL of tetraethoxyorthosilicate (TEOS) were added, and the suspension was stirred on a magnetic stirrer. A silica layer containing hydroxyl groups was formed on the MNP surface after stirring for 10 h at room temperature.

The second modification involved reacting 250 mg of TEOS-coated MNPs in 50 mL anhydrous toluene with 5 mL of 3-(trimethoxysilyl)propyl methacrylate (MPS). The reaction was carried out at room temperature under an inert atmosphere with continuous stirring for 10 h. The resulting silane-functionalized MNPs (Fe₃O₄@SiO₂-C = C) were collected magnetically, washed with ethanol and deionized water, and vacuum-dried for later use. For polymerization, 0.8 mmol of 1-vinylpyridine and 0.2 mmol of SY dye were dissolved in 30 mL of ethanol for the imprinted polymers and stirred for 3 h. Then, 200 mg of the functionalized MNPs were added, along with 4.0 mmol of EGDMA and 0.05 mmol of AIBN and the mixture was stirred for 12 h at 60 °C under nitrogen protection to ensure homogeneity^16,17^. Parallel synthesis was performed to prepare non-imprinted polymers (NIPs) without the template dye.

### Characterization

The morphological and physicochemical properties of the synthesized magnetic molecularly imprinted polymers (MMIPs) and non-imprinted polymers (MNIPs) were investigated. Surface morphology and particle structure were examined using scanning electron microscopy (SEM, JEOL JSM 5910, Akishima, Tokyo, Japan). Functional group analysis was performed via Fourier-transform infrared spectroscopy (FTIR) with an ATR-equipped Thermo Electron system (Waltham, MA, USA). Elemental composition and distribution were evaluated using X-ray fluorescence (XRF, Shimadzu RF-6000, Japan).

### Sorption experiments

Sorption experiments were performed to evaluate the binding efficiency of the synthesized MMIPs and MNIPs under controlled operational conditions. Batch adsorption studies were conducted by varying adsorbent dosage (2–10 mg), initial SY dye concentrations (5–35 ppm), pH (2–9), and contact time (3–18 min). For each experiment, the adsorbent was added to 10 mL of dye solution in glass vials and shaken at 200 rpm using an orbital shaker.

After the designated contact time, the suspensions were centrifuged at 5000 rpm for 5 min, and the clear supernatant was collected for analysis. The residual SY concentration (C_f_) was determined using a UV–Vis spectrophotometer at 490 nm. This wavelength was selected because SY exhibits its maximum absorbance (λ-max) around 482–485 nm in aqueous media; however, under our experimental pH and buffer conditions, a bathochromic shift to 490 nm was observed during preliminary wavelength scanning (200–700 nm). Therefore, all quantitative measurements were performed at 490 nm to ensure maximum sensitivity. SY concentrations were determined using calibration curves plotted with standard solutions (concentration range 5–40 ppm, R² > 0.998), based on a linear relationship between absorbance and concentration. All measurements were repeated three times.

The equilibrium adsorption capacity (*q*_e_, mg g^− 1^) was calculated using the following Eq^18^.:

$$\:{q}_{e}=\frac{{C}_{initial}-{C}_{final}}{mass\left(g\right)}\times\:Vol\left(L\right)$$

Where q_e​_ (mg g^− 1^) is the equilibrium adsorption capacity, C_initial_ and C_final_​ (mg L^− 1^) are the initial and equilibrium concentrations of the SY in solution, respectively, Vol (L) is the volume of the SY solution, and mass (g) is the mass of the adsorbent used. The vaues of C_initial_ were set experimentally, while C_final_​ was determined from the calibration curve obtained using UV-Vis spectrophotometry after equilibrium was reached.

### Electrochemical experiments

The electrochemical sensing properties of the polymers were assessed using a GAMRY Interface 1010B potentiostat in a standard three-electrode configuration. The system consisted of a platinum plate counter electrode, a silver/silver chloride **(**Ag/AgCl**)** reference electrode, and a Teflon working electrode filled with carbon paste embedded with MMIPs^19^. The working electrode had a cavity of 1.5 mm diameter and 1 mm depth.

The sensing material was prepared by mixing 85 mg graphite powder with 15 mg MMIPs in 1 mL deionized water to form a homogeneous paste, which was left to dry at room temperature for 24 h. Subsequently, 1 mL of paraffin oil was added to achieve the desired consistency. The paste was pressed into the Teflon chamber containing a platinum disc to establish electrical contact^20^.

Commercially available fruit juice samples were purchased from a local market and analyzed without further purification. Prior to analysis, the juice samples were degassed by ultrasonication for 10 min to remove dissolved gases. The samples were then filtered through a 0.45 μm membrane filter to eliminate suspended solids and pulp. To minimize matrix effects, the filtered juice was diluted tenfold with 0.1 M phosphate buffer solution (PBS, pH 7.0).

For recovery studies, known concentrations of Sunset Yellow were spiked into the diluted juice samples at three different levels. The spiked samples were gently mixed and allowed to equilibrate for 15 min at room temperature to ensure uniform distribution of the analyte.

## Results and discussion

### Morphological and structural characterization

#### SEM results

**Fig. 1:**
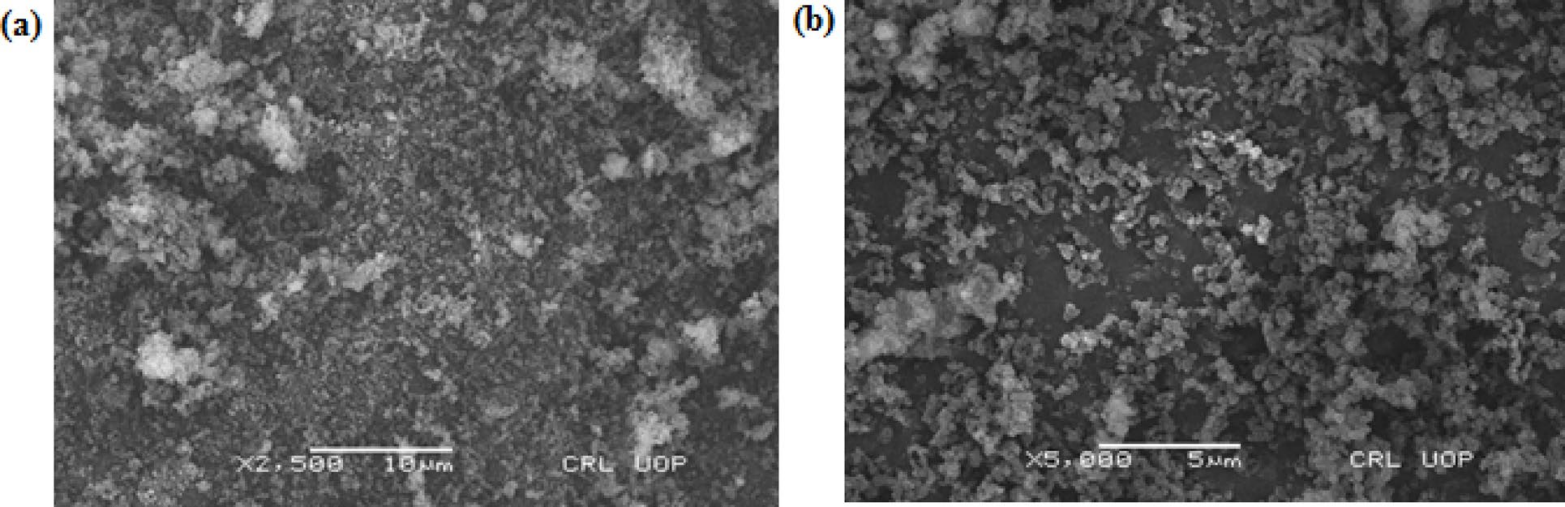
SEM analysis (**a**) MMIPs (**b**) MNIPs.

Scanning electron microscopy (SEM) revealed irregular spherical MMIPs particles with a core-shell structure (Fig. 1). Their rough texture is attributed to imprinting cavities, which generate a heterogeneous surface morphology. In contrast, MNIPs exhibited a larger pore structure and a smoother surface. MMIPs had an average particle diameter of approximately 69 nm, whereas MNIPs displayed a significantly larger average diameter of around 194 nm.

#### FTIR results

**Fig. 2:**
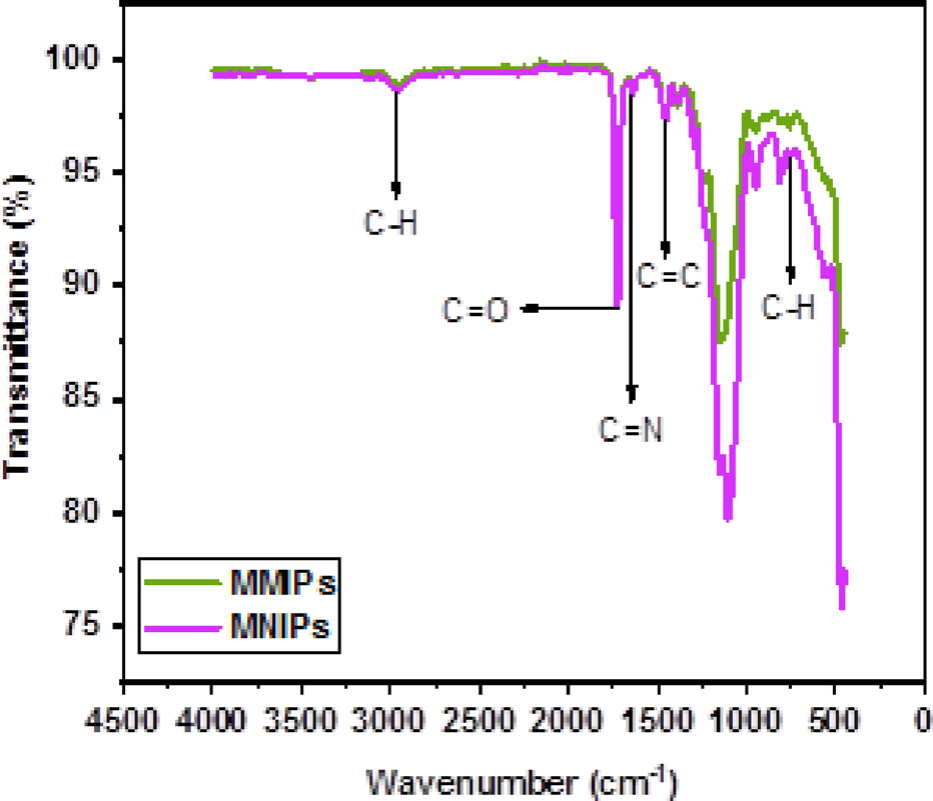
FTIR analysis of MMIPs and MNIPs.

Fourier-transform infrared spectroscopy (FTIR) confirmed the successful polymerization of MMIPs (Fig. 2). The Fe–O bond of magnetite was identified as the source of an absorption band at 532 cm^−^¹. The efficient formation of the polymers was supported by the similar spectral features displayed by both MMIPs and MNIPs^21–23^. The C = N and C = C stretching vibrations of the pyridine ring were associated with bands observed at 1638 and 1452 cm^−^¹. At 2942 cm^−^¹, a distinctive C–H stretching band appeared^24^. The C = O stretching vibration of EGDMA was also correlated with the sharp band at 1721 cm^−^¹, while the C–O stretching was indicated by a prominent peak at 1144 cm^−^¹. Additionally, the out-of-plane bending vibration of C–H was assigned to the absorption band at 751 cm^−^¹^25–27^.

#### XRF results

The XRF study was conducted to investigate the elemental composition of the synthesized product. The quantitative X-ray fluorescence (XRF) analysis of MMIPs and MNIPs is presented in Tables 1 and 2, respectively. Both silicon (Si) and iron (Fe) are clearly present in the samples, according to the spectra. The successful incorporation of magnetite (Fe₃O₄) nanoparticles into the polymer matrix is confirmed by the substantial iron content^28^. Furthermore, the significant silicon concentration provides strong evidence of surface modification with a silica layer, which was introduced to enhance the magnetite particles’ stability, functionality, and dispersion. The formation of magnetic nanoparticles and their subsequent silanization was verified by this elemental analysis^29–31^.

**Table 1:** Quantitative results of XRF analysis for MMIPs.

Analyte	Result (%)	[3-sigma]	Proc.-Calc.	Line	Int. (cps/µA)
SiO₂	65.542	[1.444]	Quan-FP	SiKa	9.9867
Fe₂O₃	32.820	[0.058]	Quan-FP	FeKa	1574.8124
SO₃	1.122	[0.067]	Quan-FP	S Ka	0.7924
K₂O	0.163	[0.020]	Quan-FP	K Ka	0.7629
MnO	0.136	[0.006]	Quan-FP	MnKa	5.8989
CaO	0.113	[0.011]	Quan-FP	CaKa	0.8607
Cr₂O₃	0.043	[0.005]	Quan-FP	CrKa	1.5958
ZnO	0.030	[0.003]	Quan-FP	ZnKa	1.6946
CuO	0.030	[0.004]	Quan-FP	CuKa	1.3943

**Table 2:** Quantitative results of XRF analysis for MNIPs.

Analyte	Result (%)	[3-sigma]	Proc.-Calc.	Line	Int. (cps/µA)
SiO₂	85.321	[1.078]	Quan-FP	SiKa	9.8804
Fe₂O₃	13.629	[0.025]	Quan-FP	FeKa	476.2846
SO₃	0.490	[0.066]	Quan-FP	S Ka	0.2110
K₂O	0.317	[0.010]	Quan-FP	K Ka	0.8787
CaO	0.134	[0.009]	Quan-FP	CaKa	0.5968
MnO	0.077	[0.003]	Quan-FP	MnKa	2.2842
V₂O₅	0.023	[0.004]	Quan-FP	V Ka	0.2797
Cr₂O₃	0.010	[0.003]	Quan-FP	CrKa	0.2172

### Binding experiments

#### Effect of pH

**Fig. 3:**
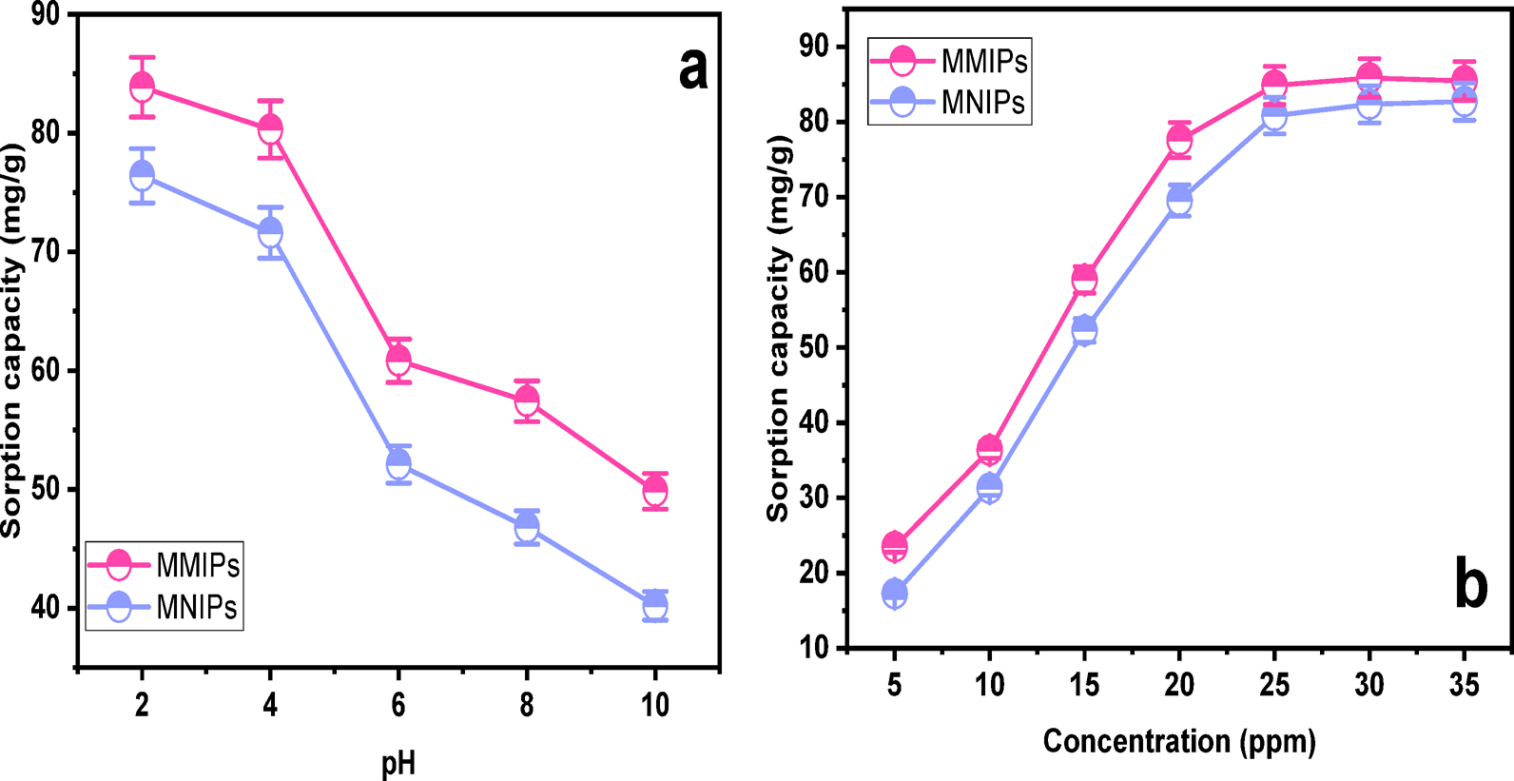
(**a**) Effect of pH on sorption efficiency of MMIPs and MNIPs (**b**) Effect of concentration on sorption efficiency of MMIPs and MNIPs.

The influence of pH on the sorption performance of both MMIPs and MNIPs was systematically investigated due to its critical role in modulating the ionization state of dye molecules and the surface charge of the sorbents (Fig. 3a). The influence of pH on the adsorption performance of MMIPs and MNIPs was evaluated over a pH range of 2–10, using a fixed initial dye concentration of 25 ppm and an adsorbent mass of 30 mg in 10 mL of solution. The results indicated that the maximum sorption capacity was achieved at an acidic pH of around 2. Sorption efficiency decreased as the pH shifted toward more basic values. This trend suggests that the polymer surface becomes protonated under acidic conditions, enhancing its interaction with the target analyte^32^.

Specifically, electrostatic interactions appear to be the main driving force behind the binding affinity of SY dye to MMIPs synthesized using 1-vinylpyridine as the functional monomer. The nitrogen atom in the pyridine ring of 1-vinylpyridine becomes protonated in acidic media, conferring a positive charge to the polymer surface^33^. Since SY is an anionic dye containing sulfonate groups, it carries a negative charge in aqueous solution. This charge complementarity enables strong electrostatic attraction between the dye molecules and the positively charged binding sites on the MMIPs surface. Consequently, these interactions significantly enhance the overall sorption efficiency of the imprinted polymer in acidic environments, resulting in a marked increase in dye binding affinity^34^.

#### Effect of concentration

Another crucial factor strongly influencing the sorption behavior of the polymers is the analyte concentration. Up to a certain threshold, an increase in sorption capacity was observed with rising SY dye concentration, as shown in Fig. 3b. The sorption capacity reached a plateau beyond this point, indicating that the accessible binding sites on the polymer surface were saturated^35^. Once saturation was achieved, additional increases in dye concentration did not enhance sorption. Based on this pattern, a concentration of 25 ppm was selected as the optimal amount for further studies. Although MMIPs exhibits a higher overall adsorption capacity than MNIPs, the similar adsorption trends observed in Fig. 3a and b suggest that the binding mechanism is largely controlled by nonspecific interactions. In addition to imprinting effects, the higher absorption of MMIPs may also be influenced by structural factors.

**Fig. 4:**
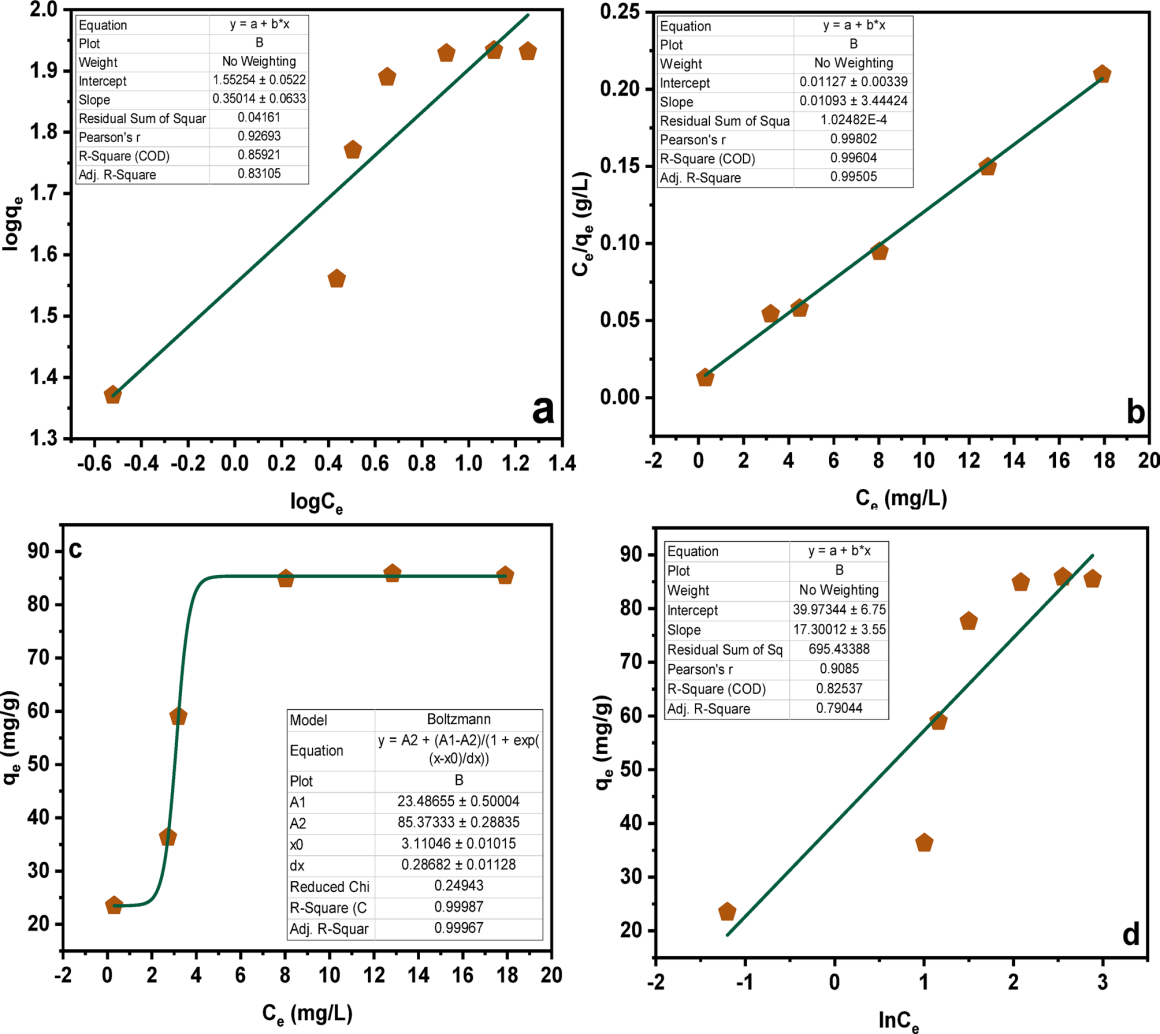
Isotherm study (**a**) Freundlich-Isotherms (**b**) Langmuir (linear)-Isotherms (**c**) Langmuir-isotherms (**d**) Temkin-isotherms.

Sorption isotherm models were applied to the equilibrium adsorption data of MMIPs to characterize the interaction mechanism between SY and the polymeric binding sites. The fitted curves for all models are shown in Fig. 4(a–d). Among the tested models, the Langmuir isotherm yielded the highest regression coefficient for the MMIPs (Table 3), suggesting the presence of strong, high-affinity binding sites capable of forming a near-monolayer coverage under the studied concentration range. However, because molecularly imprinted polymers inherently possess heterogeneous binding sites, the Langmuir model alone does not fully represent their sorption behavior^36–38^.

**Table 3:** Sorption isotherm parameters of SY onto molecularly imprinted polymers.

Isotherms	Parameters	Values
Freundlich Isotherm	N	2.8563 L/g
	K_F_	718.29 mg/g
	R^2^	0.8592
Langmuir Isotherm	K_L_	88.731 L/g
	a_L_	0.9698 L/mg
	q_o_	91.492 mg/g
	R^2^	0.996
Temkin Isotherm	B_T_	3.546 mg/g
	A_T_	1.8737 L/g
	R^2^	0.825

To address this, the Freundlich and Temkin models were also evaluated to describe surface heterogeneity and adsorbate–adsorbent interaction energy, respectively. In addition, the Langmuir–Freundlich (Sips) model, frequently used for MIP systems, was included to account for non-uniform site energy distributions through its heterogeneity exponent (n). This hybrid model combines the monolayer capacity of the Langmuir equation with the heterogeneity of the Freundlich model, providing a more realistic description of adsorption on imprint-generated cavities. For the MMIPs, the Sips model produced a heterogeneity factor (*n* < 1), confirming the presence of multiple classes of binding sites.

A brief description of the main parameters used in each model is provided for clarity. In the Freundlich model, K_f_ represents adsorption capacity and n reflects surface heterogeneity. In the Langmuir model, K_L_ is the affinity constant and q₀ is the theoretical monolayer adsorption capacity. For the Temkin model, B_T_ relates to adsorption heat and A_T_ represents the binding constant. The Langmuir–Freundlich model incorporates the Langmuir capacity (q₀) along with the heterogeneity parameter n, making it particularly suitable for characterizing real MIP systems^38^. Table 3 shows the adsorption parameters of MMIPs under the studied conditions.

#### Effect of time

**Fig. 5:**
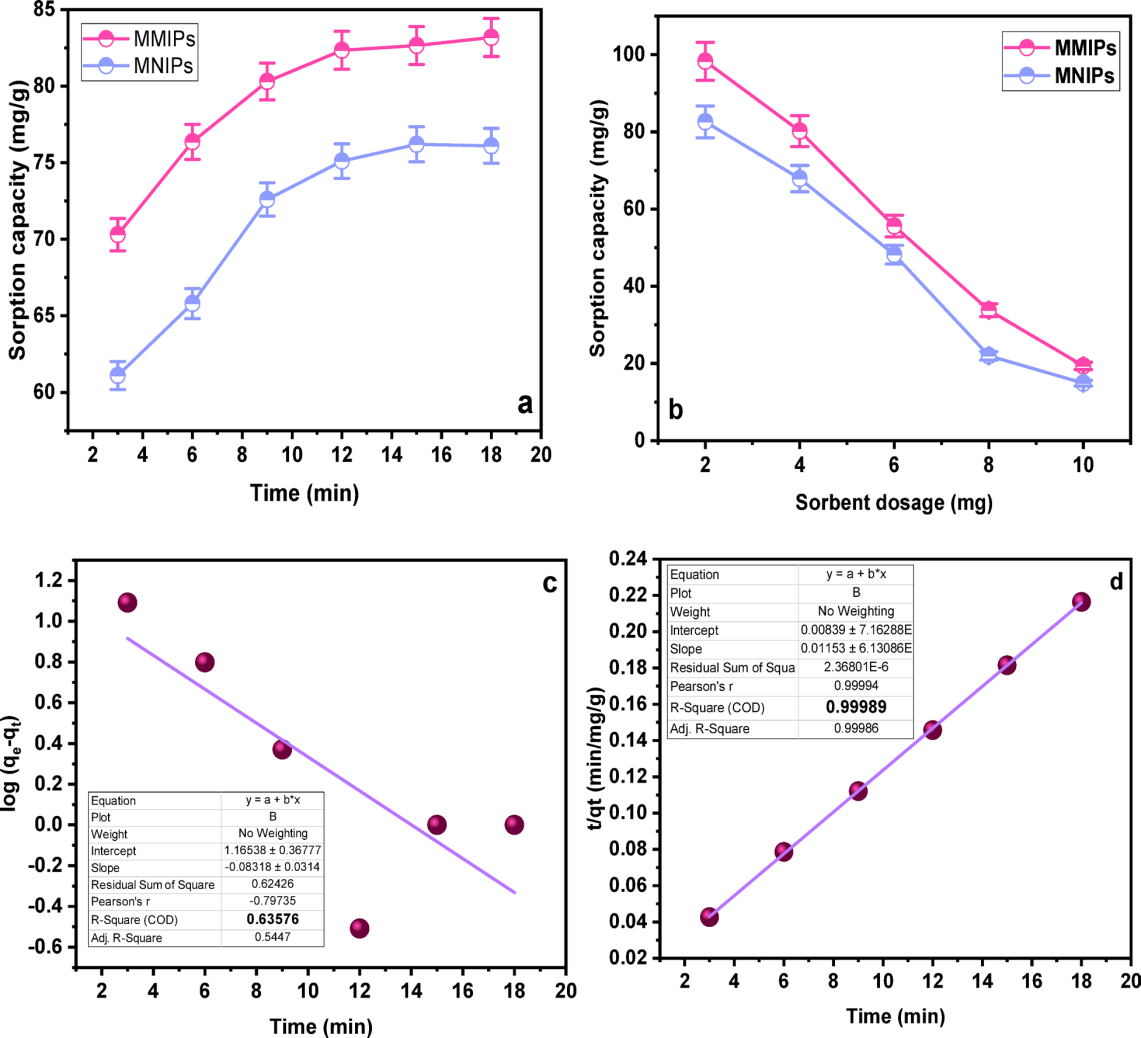
(**a**) Effect of Time on sorption efficiency of MMIPs and MNIPs, (**b**) Effect of sorbent dosage on sorption efficiency of MMIPs and MNIPs, (**c**) First-order kinetic study, (**d**) Second-order kinetic study.

Time is another important factor significantly influencing the overall sorption performance (Fig. 5a). This was investigated by examining the effect of contact time on the sorption efficiency of the three types of MMIPs over a period of 3 to 18 min. Initially, the sorption capacity increased with time, indicating active interaction between the dye molecules and the accessible binding sites^39^. However, the sorption rate eventually plateaued, suggesting that the sorbent had reached saturation and all active sites were occupied. The influence of sorbent dosage on the sorption efficiency was also evaluated, showing that an optimum dosage exists to maximize dye removal (Fig. 5b).

The experimental data were analyzed using the pseudo-first-order and pseudo-second-order kinetic models to gain deeper insight into the sorption process (Fig. 5c and d)^40^. The fitting results showed that the pseudo-second-order model best described the sorption kinetics, with a high correlation coefficient (R² = 0.9998) (Table 4). According to this model, the rate-limiting step is chemisorption, where the sorption process depends on both the interaction between sorbent and sorbate and the availability of active sites. Specifically, the model indicates that the sorption rate is directly proportional to the number of active binding sites^41^.

**Table 4:** Kinetic parameters for SY sorption on MMIPs using Pseudo-First-Order and Pseudo-Second-Order models.

First-order kinetics	
K	-0.191563
q_e_	14.635
R^2^	0.6357
**Second-order Kinetics**	
q_e_	86.7302
K_2_	0.01584
R^2^	0.99989

For SY dye, electrostatic interactions are the main driving force behind sorption. Strong ionic interactions occur between the dye molecules and the protonated functional groups of the polymer once the dye diffuses from the bulk solution to the MMIPs’ surface. These results demonstrate that sorption is not only effective but also highly dependent on the strength of contact between the interacting species^42^.

K and qₑ in the pseudo-first-order model represent the rate constant and the equilibrium sorption capacity, respectively, while qₑ and K₂ in the pseudo-second-order model represent the equilibrium sorption capacity and the rate constant. R² indicates the regression coefficient, reflecting the goodness of fit for each kinetic model.

#### Effect of sorbent dosage

The sorption efficiency of MMIPs is largely dependent on the amount of sorbent used. Different dosages ranging from 2 to 10 mg (0.002–0.01 g) were evaluated. The maximum sorption capacity was observed at a dose of 2 mg (Fig. 5b]. Performance decreased when the dosage exceeded this value. Consequently, 2 mg was determined to be the optimal dosage for further experiments^43^.

### Electrochemical studies

**Fig. 6:**
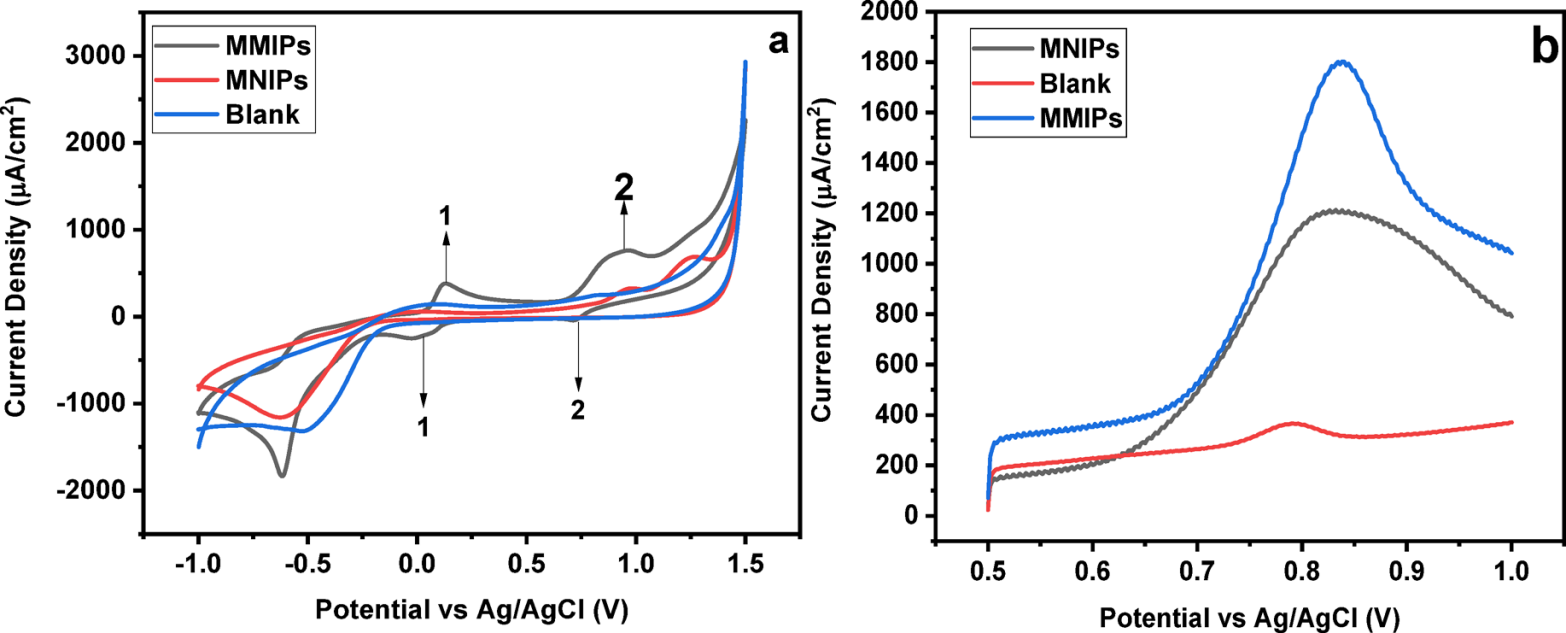
Comparison (**a**) CV for MMIPs, MNIPs, and Blank (**b**) SWAdASV for MMIPs, MNIPs, and Blank.

Cyclic voltammetry (CV) and Square Wave Adsorptive Anodic Stripping Voltammetry (SWAdASV) were performed to investigate the electrochemical sensing capabilities of MMIPs and MNIPs. Comparison among MMIPs, MNIPs, and a blank sample (without dye) revealed that MMIPs exhibited a significantly higher response (Fig. 6a and b]. This enhanced performance is attributed to the presence of selective cavities on the sensor surface, which enable dye pre-concentration, as evidenced by a pronounced oxidation peak^44^. Distinct redox peaks were particularly evident with MMIP-based sensors. The main oxidation peak (peak 2) exhibited a current density of 755.64 µA/cm² at a potential of 0.936 V, showing a sharper response than that observed for MNIPs or the blank. The SWAdASV data (Fig. 6b) followed a similar trend, with MMIPs producing the highest peak current of 1802 µA/cm², compared to 1205 µA/cm² for MNIPs and 366.2 µA/cm² for the blank. These results demonstrate that MMIPs possess higher sensitivity toward SY dye due to their molecularly imprinted recognition sites^45^.

Mechanistically, the enhanced performance of MMIPs is attributed to specific interactions between SY and the functional monomer 1-vinylpyridine (1-VP). The sulfonate groups of SY interact electrostatically with protonated pyridine groups, and the interaction is further stabilized by hydrogen bonding and π–π stacking. Complementary cavities retaining these functionalities after template removal allow rapid and selective rebinding. The stronger and more intense oxidation peaks observed for MMIPs can be explained by this preconcentration effect. Overall, MMIPs prepared with 1-VP provide accessible, high-affinity sites that significantly enhance the electrochemical detection of SY, offering a sensitive and selective platform for monitoring azo dyes.

#### Effect of pH

**Fig. 7:**
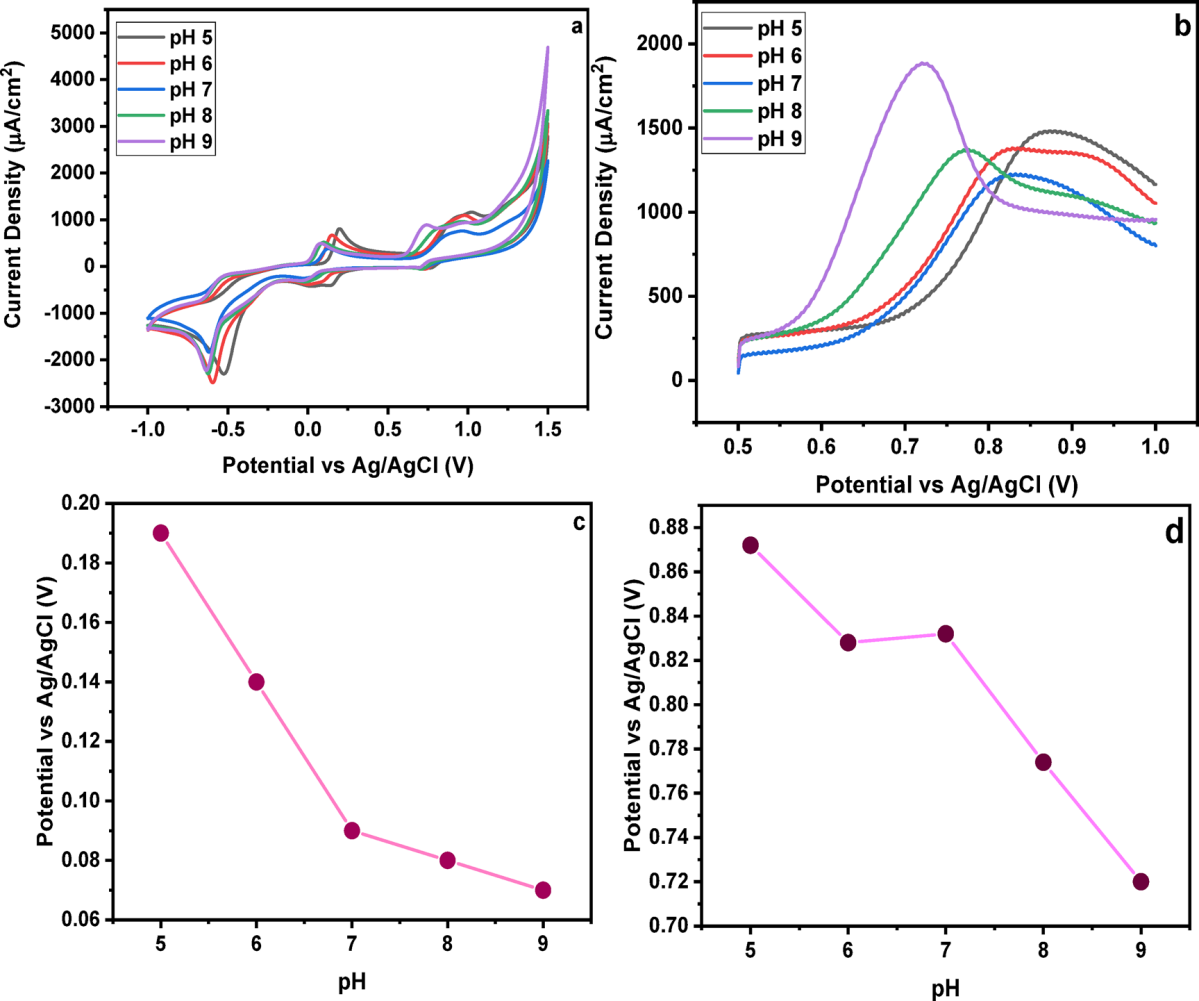
Effect of pH on the detection response of SY dye (**a**) CV (**b**) SWAdASV (**c**) peak-potential (CV) (**d**) peak-potential (SWAdASV).

The pH of the surrounding medium plays a crucial role in determining the electrochemical behavior of SY. Cyclic voltammetry (Fig. 7a) showed that variations in pH caused clear shifts in the oxidation peak potential, with the peak progressively moving toward more negative values as the pH increased. A linear correlation between anodic peak potential (Epa) and pH was observed, indicating proton involvement in the oxidation process. Among the tested conditions, the highest and most well-defined response was recorded at pH 7, which exhibited both the largest peak area and the lowest peak potential^46^.

The enhanced signal at pH 7 is attributed to the favorable protonation state of the SY molecule and the 1-vinylpyridine units in the polymer, which together facilitate improved electron transfer and stronger interactions within the sensing interface. This near-neutral environment avoids excessive protonation at low pH and insufficient proton availability at high pH, resulting in improved charge-transfer kinetics^47^.

These observations were further supported by SWAdASV measurements (Fig. 7b), where the stripping current also reached a maximum at pH 7, confirming that this condition favors efficient analyte accumulation and electrochemical oxidation. At lower pH, over-protonation of SY and the pyridine groups diminished interaction efficiency, whereas at higher pH, reduced proton involvement weakened the proton-coupled oxidation process (Fig. 7c and d). Based on these combined results, pH 7 was selected as the optimal working pH for subsequent electrochemical sensing studies^48^. These results highlight that maintaining an optimal pH not only maximizes electrochemical response but also ensures efficient and selective recognition of SY by the molecularly imprinted sites.

#### Effect of time

**Fig. 8:**
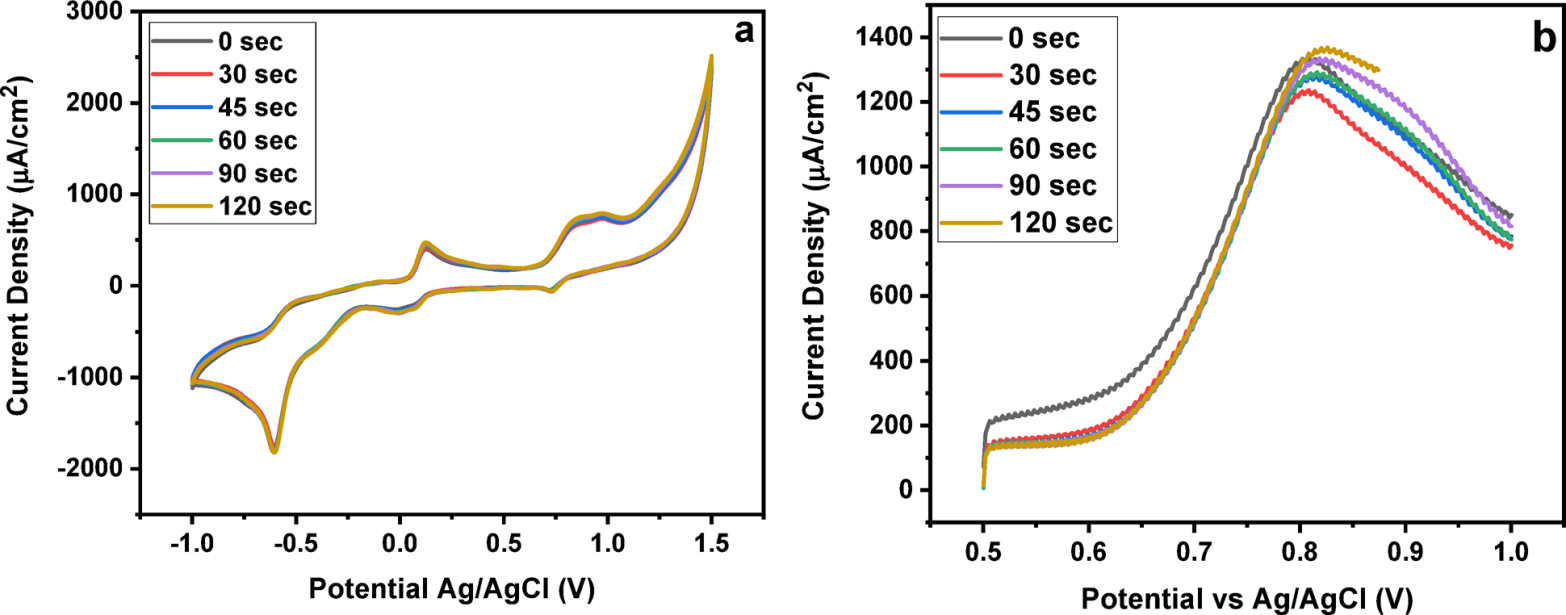
Effect of time (**a**) CV (**b**) SWAdASV.

The effects of accumulation time and applied potential on the electrochemical response of SY were evaluated using both MMIP- and MNIP-modified electrodes (Fig. 8a, b). Accumulation times ranged from 30 to 120 s. In CV measurements on the MMIPs electrode, increasing the accumulation time beyond 90 s produced only marginal increases in peak current, indicating that the available binding sites on the electrode surface were approaching saturation. SWAdASV measurements showed a similar trend: the peak current increased rapidly at short accumulation times and then plateaued at longer times, reflecting the maximum occupancy of active binding sites. In comparison, the MNIP-modified electrode exhibited lower peak currents and a less pronounced saturation effect under identical conditions, consistent with nonspecific adsorption. Based on these observations, an accumulation time of 90 s was selected as optimal for subsequent electrochemical measurements, balancing high signal intensity and preventing unnecessary saturation of the MMIPs binding sites^49–51^.

#### Effect of concentration

**Fig. 9:**
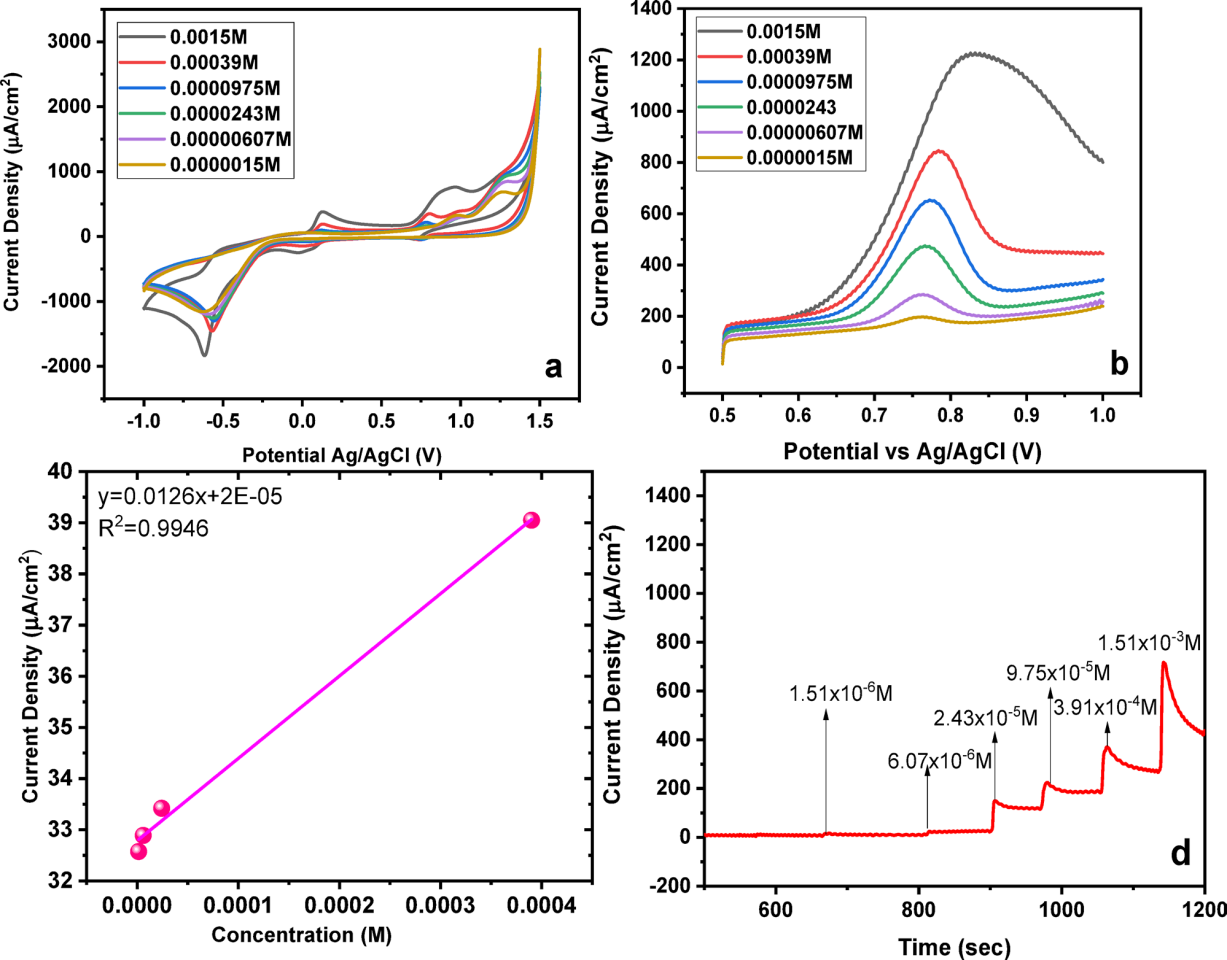
Concentration effect (**a**) CV-response (**b**) SWAdASV response (**c**) Calibration curve (**d**) Chronoamperometry test.

Figure 9(a-d) illustrates how the electrochemical response of MMIP-based sensors is influenced by varying the concentration of SY over a range of 1.51 × 10⁻⁶ to 1.51 × 10⁻³ mol L⁻¹. The highest current density for both CV and SWAdASV was observed at 1.5 × 10⁻³ M, which was therefore selected for further experiments. A calibration plot of current density versus concentration exhibited a strong linear relationship, with a correlation coefficient (R²) of 0.9946 (Fig. 9c)^52^.

Chronoamperometric analysis was conducted to investigate the diffusion behavior of SY in more detail. Measurements were performed for concentrations ranging from 0.0000015 to 0.0015 M. Under a constant applied potential of 1.18 V, the data were plotted with time (seconds) on the x-axis and current density (µA/cm²) on the y-axis (Fig. 9d)^53^. The chosen potential corresponds to the anodic oxidation peak of SY obtained from the optimized voltammetric studies (CV). Operating the chronoamperometric measurement at this peak potential ensures maximum faradaic response, high sensitivity, and minimal contribution from non-faradaic background current. These indicate that an optimal concentration ensures maximum sensor response while maintaining linearity for quantitative detection.

#### Interference study

**Fig. 10:**
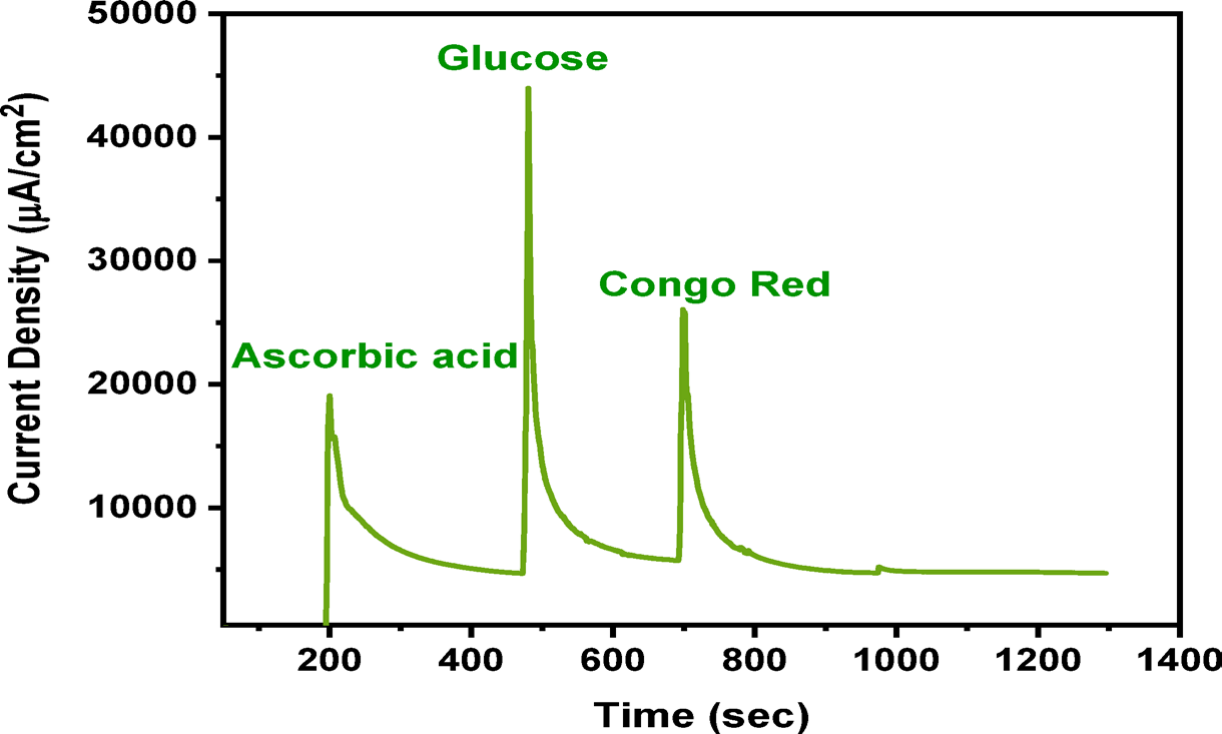
Interference study of SY detection by MMIP sensors.

An interference study was carried out to assess the selectivity of the MMIP-based magnetosensor toward SY under the optimized detection conditions. Cyclic voltammetry (CV) was employed for all measurements. A fixed concentration of SY (10 µM) was used, while each potential interfering species, ascorbic acid, glucose, and Congo Red dye, was added at a 10-fold higher concentration (100 µM) to evaluate their influence on the sensor response (Fig. 10). All experiments were performed using the same MMIP-modified magnetosensor, which was thoroughly rinsed and reconditioned between successive measurements to maintain consistency^54^.

The introduction of these interferents caused only minor variations in the oxidation peak current of SY, indicating negligible interaction with the imprinted binding sites. Since the presence of these compounds did not significantly affect the sensor performance, the results confirm the high selectivity of the MMIP-based magnetosensor for SY.

#### Real sample analysis

Water samples collected from local rivers and wastewater treatment plants were analyzed to assess the practical applicability of the MMIP-based sensor. Prior to analysis, samples were filtered to remove suspended particulates and then spiked with known concentrations of SY at three levels (low, medium, and high). These spiking levels were selected based on (i) the linear working range of the sensor, ensuring measurements within the most sensitive region of the calibration curve, and (ii) the concentration range typically reported for synthetic dyes in environmental water samples. As presented in Table 5, the recoveries obtained ranged from 72.9 to 99.3%, confirming the high accuracy and reproducibility of the sensor in complex matrices. Overall, the MMIP-based sensor demonstrates strong potential for reliable SY detection in real water and beverage samples, supporting its suitability for practical environmental and food analysis.

**Table 5:** Real sample analysis for SY using MMIP-based sensors.

Sample	Added	Found	Recovery
River water	7.01 × 10^− 6^M	(5.94 ± 0.03) × 10^− 6^M	84.7%
Industrial water	4.01 × 10^− 4^M	(3.98 ± 0.04) × 10^− 4^M	99.3%
Juice	3.43 × 10^− 5^M	(2.5 ± 0.06) × 10^− 5^M	72.9%

To comprehensively evaluate the analytical performance of the MMIP-based electrochemical sensor, a series of validation experiments was conducted. The sensor displayed a clear concentration-dependent increase in peak current, yielding a linear calibration curve within the tested range. The LOD and LOQ, calculated using the 3σ/slope and 10σ/slope criteria, were 5.82 × 10^− 5^ M and 1.76 × 10^− 4^ M, respectively.

## Conclusion

An electrochemical sensor based on magnetic molecularly imprinted polymers (MMIPs) was successfully developed for the selective detection of SY. Magnetic nanoparticles enhanced the surface area and facilitated rapid separation, while the imprinted polymer layer provided highly specific recognition sites complementary to the target dye. The sensor exhibited excellent sensitivity, selectivity, and reproducibility across a wide concentration range, with a low detection limit suitable for trace-level monitoring. Optimal performance was achieved at pH 7, an accumulation time of 90 s, and a concentration of 1.5 × 10⁻³ M, ensuring efficient pre-concentration and maximal electrochemical response. In real sample analyses, the sensor demonstrated high recovery rates ranging from 72.9 to 99.3**%**, confirming its accuracy and reliability in complex matrices such as river water, industrial effluents, and beverages. These results highlight the sensor’s robust stability and practical applicability for environmental monitoring and food safety assessment. Overall, the findings underscore the potential of MMIP-based platforms as effective tools for the accurate and rapid detection of artificial food colorants. This strategy can be extended to other hazardous additives or emerging contaminants, paving the way for the development of advanced molecularly imprinted electrochemical sensors with broad analytical and environmental applications. In the future, we plan to perform quantitative comparisons with NIP-derived parameters to provide a more comprehensive assessment of the imprinting effect. Correspondingly, a systematic adsorption study of MNIPs will be performed under the same experimental conditions to enable direct comparison.

## Data Availability

The datasets used and/or analysed during the current study are available from the corresponding author on reasonable request.

## Declarations

### Limitations of the study

The current study did not encounter any significant limitations. All experimental objectives were successfully achieved, and the results are considered robust and reliable within the designed scope of the research.
